# Epidemiology of microbiological findings in the lower respiratory tract in mechanically ventilated patients with and without inhalation injury

**DOI:** 10.3389/fcimb.2026.1789404

**Published:** 2026-05-13

**Authors:** Terezie Polackova, Katerina Vyklicka, Bretislav Lipovy, Jan Bohm, Filip Raska, Iva Tresnerova, Martin Hladik, Iva Kocmanova, Marketa Hanslianova, Petra Borilova Linhartova

**Affiliations:** 1 RECETOX, Faculty of Science, Masaryk University, Brno, Czechia; 2 Department of Burns and Plastic Surgery, University Hospital Brno, Brno, Czechia; 3 Department of Burns Medicine, Third Faculty of Medicine, Charles University and University Hospital Kralovske Vinohrady, Prague, Czechia; 4 Department of Clinical Microbiology and Immunology, University Hospital Brno, Brno, Czechia; 5 Department of Microbiology, Vyskov Hospital, Vyskov, Czechia

**Keywords:** burns, colonization, inhalation injury, pathogen, survival analysis

## Abstract

**Background:**

In mechanically ventilated burn patients, inhalation injury may increase the risk of lower respiratory tract colonization or infection with potentially pathogenic microorganisms and worsen in-hospital outcomes.

**Objective:**

We investigated the occurrence of potentially pathogenic microorganisms in lower respiratory tract samples and their association with in-hospital mortality in mechanically ventilated patients with and without inhalation injury.

**Methods:**

We conducted a retrospective observational study at a tertiary burn center (2007–2022) including patients who required mechanical ventilation for ≥2 days and sustained cutaneous burns and/or inhalation injury; clinical and microbiological data were extracted from medical records.

**Results:**

Among 311 mechanically ventilated patients, 305 had cutaneous burns, 168 had inhalation injury, and 180 had lower respiratory tract samples positive for potentially pathogenic microorganisms. Gram-positive bacteria predominated early during hospitalization, while gram-negative bacteria and fungi became more frequent over time. Patients with inhalation injury showed shorter time to in-hospital death than those without inhalation injury (mean 32 vs 64 days), regardless of total body surface area burned. Detection of extended-spectrum beta-lactamase–producing organisms was associated with higher mortality risk and substantially longer hospital stays.

**Conclusion:**

Inhalation injury was associated with a higher likelihood of lower respiratory tract positivity for potentially pathogenic microorganisms and with increased in-hospital mortality in mechanically ventilated patients.

## 1 Introduction

Inhalation injury (INHI) is defined as acute airway injury and mucosal damage caused by inhalation of toxic products of combustion or hot steam (Jones et al., 2017). This type of injury can also occur as a result of irritation of the airways by a variety of dangerous chemicals. The estimated prevalence of INHI among burn patients varies, with observed prevalence ranging from 7 to 36% (Palmieri, 2007; You et al., 2014; Tracy et al., 2020; Charles et al., 2022). In the care of burn patients, the median survival time of patients with INHI was significantly shorter than that of patients without this injury (Charles et al., 2022). Overall, INHI significantly increased morbidity and mortality of such patients by 15 to 45% (Cancio, 2005; Rehberg et al., 2009; Woodson, 2009; Walker et al., 2015; Walsh et al., 2017; Dyamenahalli et al., 2019). Other factors associated with mortality include burn size and age (Walker et al., 2015; Charles et al., 2022) – for example, patients with more extensive burns are more likely to suffer from INHI than those with lower burn extent (Tredget et al., 1990).

Early-onset complications following INHI include direct mucosal damage to the respiratory tract, mucosal sloughing, and the initiation of inflammatory processes, all of which results in severe airway obstruction and compromises gas exchange. Smoke-related toxic compounds can further damage the epithelial and capillary endothelial cells of the airway. Mucociliary transport is disrupted and, therefore, the clearance of microorganisms is reduced. This, along with the necrosis of respiratory epithelium, predisposes patients with INHI to secondary bacterial and fungal invasion and superimposed tracheobronchitis and bronchopneumonia (Jones et al., 2017; Johnson et al., 2025).

The patients are exposed to microorganisms ubiquitous in the hospital environment that can colonize damaged mucous membranes of the respiratory tract. Unfortunately, microorganisms associated with hospital environments exhibit higher antimicrobial resistance and are dangerous sources of nosocomial infections associated with prolonged length of hospitalization (Liodaki et al., 2014).

The development of a lower respiratory tract infection (LRTI) is one of the most serious complications in patients with INHI. Generally, when the infection spreads, commensal bacterial species are displaced by potentially pathogenic microorganisms (Lipový et al., 2016; Pu et al., 2020), particularly bacteria. The PPMs most frequently detected in the lower respiratory tract (LRT) of patients with INHI include *Staphylococcus aureus*, *Pseudomonas aeruginosa*, and *Acinetobacter baumannii* (Cen et al., 2015). Moreover, microbial shifts from gram-positive (G+) to gram-negative (G-) species in the LRT typically occurs within the first week(s) of hospitalization (Lachiewicz et al., 2017; Holoubek et al., 2018; Corcione et al., 2020), accompanied with the reduction of microbiome diversity and overdevelopment of resistant strains of PPMs, such as extended-spectrum beta-lactamase (ESBL+) producers. Close microbiological surveillance and prompt response to all changes, together with targeted antimicrobial therapy, are then the basic prerequisites for the successful treatment of these patients. Patients with INHI also typically require mechanical ventilatory support, which, however, given the vulnerability of the already damaged respiratory tract, further increases the risk of developing LRTI.

Inconsistencies in INHI diagnosis and treatment approaches may lead to differences in the reported prevalence of INHI. Therefore, distinguishing the specific effects of various causes of INHI is challenging. Hence, we aimed i) to create a comprehensive overview of PPMs identified in the LRT of patients, ii) to evaluate the association of dynamics of microbiological findings with the presence/absence of INHI in these patients and iii) to evaluate the incidence of ESBL producers.

## 2 Materials and methods

### 2.1 Study design and clinical data

The study was approved by the Ethics Committee of the University Hospital Brno, Czech Republic (No. 04-120220/EK, date 12/FEB/2020). Patients hospitalized at the Department of Burns and Plastic Surgery of the University Hospital Brno (Burn Centre) between 2007 and 2022 were included in this retrospective observational study. Patients with or without INHI who were mechanically ventilated for at least two days were included in the study. The diagnosis of INHI was established based on clinical symptoms (dyspnea, cough, dysphonia, presence of ash or soot in the upper respiratory tract) and confirmed by bronchoscopy or laryngoscopy (Jones et al., 2017).

Of clinical parameters, the presence of INHI and the extent of the burns (total burn surface area, TBSA) were collected from the records, along with the number of procedures performed under general anesthesia, duration of hospitalization, mortality, place of intubation (out-of-hospital vs. in-hospital), full thickness burn area (FTBA), the Abbreviated Burn Severity Index (ABSI), burns to the neck or head, duration of mechanical ventilation, and tracheostomy.

Additionally, basic biochemical parameters (glucose and albumin levels) were measured throughout the patients’ hospitalization. A detailed medical history was taken, including comorbidities such as diabetes, asthma, and chronic obstructive pulmonary disease. Demographic data were also recorded.

### 2.2 Microbiological data

Tracheal aspirate and bronchoalveolar lavage were the predominant materials collected from the LRT. Processing of these biological materials followed the Standard Operating Procedures of the Microbiology Department of the University Hospital Brno, Czech Republic. All material from the LRT was inoculated onto basic culture media – blood agar and MacConkey agar – as well as onto special culture media, namely: VL anaerobic blood agar (for anaerobic culture), chocolate agar (enriched with nutrients derived from erythrocyte lysis for better growth of more demanding microorganisms, such as the *Haemophilus* sp.) and blood agar with added NaCl (for better growth of *Staphylococcus* sp.).

Samples on blood agar, blood agar supplemented with NaCl, and chocolate agar were incubated for 18–24 hours in an atmosphere with increased CO_2_ level, MacConkey agar plates were incubated for 18–24 hours in a standard atmosphere, and the VL anaerobic blood agar plates were incubated for 48 hours in an anaerobic atmosphere. All cultures were incubated at 37 °C and examined after the aforementioned periods. Colonies visible on the agars were identified mostly using matrix-assisted laser desorption-time of flight (MALDI-TOF, Bruker, Germany) but, depending on the type of microorganisms (such as viridans streptococci), some were identified through API tests (BioMérieux, France).

Antibiotic susceptibility was tested using the disk diffusion method, supplemented in some cases by the determination of Minimum Inhibitory Concentration (MIC) values using the European Committee on Antimicrobial Susceptibility Testing (EUCAST) interpretative criteria.

The occurrence of infectious diseases in the LRT can be generally classified into two clinically significant phases of microbial infection (Sarabahi et al., 2012), namely the period within the first two days of hospital admission and ≥5 days after admission. Pathogens identified during the first two days are generally considered community-acquired and typically involve strains without multidrug resistance. On the other hand, PPMs detected only after Day 5 of hospitalization are usually regarded as hospital-acquired. To be able to observe the microbial dynamics throughout hospitalization, we have analyzed LRT samples from more periods, namely: i) within the first two days of hospitalization, ii) between Days 3 and 5, iii) between Days 6 and 10, iv) between Days 11 and 15, and v) after Day 15 of hospitalization.

### 2.3 Statistical analysis

Differences between groups were compared using odds ratios (OR) with confidence intervals (CI), the Friedman test, t-test, Mann-Whitney test, Fisher’s exact test, or Pearson’s chi-squared test as appropriate. To analyze survival time, the cumulative incidence function and the Kaplan-Meier estimate were used. Log-rank test was used to compare the time-to-event distributions between patient groups. For the analysis of in-hospital mortality, a Cox proportional hazards model with a time-dependent exposure was additionally used to assess the association between the occurrence of the first PPM+ during hospitalization and the subsequent hazard of in-hospital death. PPM+ was modeled as a time-varying covariate using start–stop interval data. Because individual patients could contribute multiple intervals, robust standard errors clustered at the patient level were applied; ties were handled using the Efron method. The final model included PPM+ status, age, sex, TBSA, and INHI, with women as the reference category for sex. Potential collinearity among the baseline covariates included in the model was assessed at the patient level using correlation analysis, group comparisons according to inhalation injury status, and generalized variance inflation factors (GVIF). The proportional hazards assumption was evaluated using Schoenfeld residual-based tests and residual plots. Influence was assessed using patient-level DFBETAS, and a sensitivity analysis excluding patients with maximum absolute DFBETAS > 0.3 was performed as a robustness check. In all analyses, *p*-values *p* < 0.05 were considered statistically significant. The data were analyzed in R (version 4.0.5) using the packages survminer, survival, gtsummary, cmprsk, ggplot2, ggsurvfit, tidycmprsk, car, dplyr, tidyr, **Table 1**, ComplexUpset, and epitools.

**Table 1:** Patient demographics and comparisons between patients without and with inhalation injury.

Variables	Patients without INHI (N = 143)	Patients with INHI (N = 168)	*p*-value	Overall (N = 311)
Sex				
Female	31 (21.7%)	37 (22.0%)	>0.999^a^	68 (21.9%)
Male	112 (78.3%)	131 (78.0%)		243 (78.1%)
Age (years)				
Median [min, max]	41.0 [17.0, 94.0]	46.0 [19.0, 92.0]	0.834^b^	45.0 [17.0, 94.0]
Length of hospitalization (days)				
Median [min, max]	37.0 [2.0, 153.0]	29.5 [2.0, 184.0]	>0.999^b^	34.0 [2.0, 184.0]
TBSA (%)				
Median [min, max]	21.0 [3.0, 75.0]	21.5 [0, 95.0]	>0.999^b^	21.0 [0, 95.0]
Full thickness burn area				
No	11 (7.7%)	14 (8.3%)	>0.999^a^	25 (8.0%)
Yes	131 (91.6%)	153 (91.1%)		284 (91.3%)
Missing	1 (0.7%)	1 (0.6%)		2 (0.7%)
Diabetes mellitus				
No	130 (90.9%)	154 (91.6%)	0.834^a^	284 (91.3%)
Yes	10 (7.0%)	7 (4.2%)		17 (5.5%)
Missing	3 (2.1%)	7 (4.2%)		10 (3.2%)
Chronic obstructive pulmonary disease (copd) or asthma				
No	136 (95.1%)	153 (91.1%)	0.834^c^	289 (92.9%)
Yes	4 (2.8%)	8 (4.8%)		12 (3.9%)
Missing	3 (2.1%)	7 (4.2%)		10 (3.2%)
Death				
No	126 (88.1%)	139 (82.7%)	0.834^a^	265 (85.2%)
Yes	17 (11.9%)	29 (17.3%)		46 (14.8%)
Burns				
No	0	6 (3.6%)	NA	6 (1.9%)
Yes	143 (100.0%)	162 (96.4%)		305 (98.1%)

INHI, inhalation injury; LRT, lower respiratory tract; TBSA, total burned surface area; PPMs, potential pathogenic microorganisms; NA, not applicable – the assumptions for applying the test were not met.

^a^ Pearson’s chi-squared test.

^b^ Mann–Whitney test.

^c^ Fisher’s exact test.

## 3 Results

### 3.1 Cohort summary

**Figure 1:**
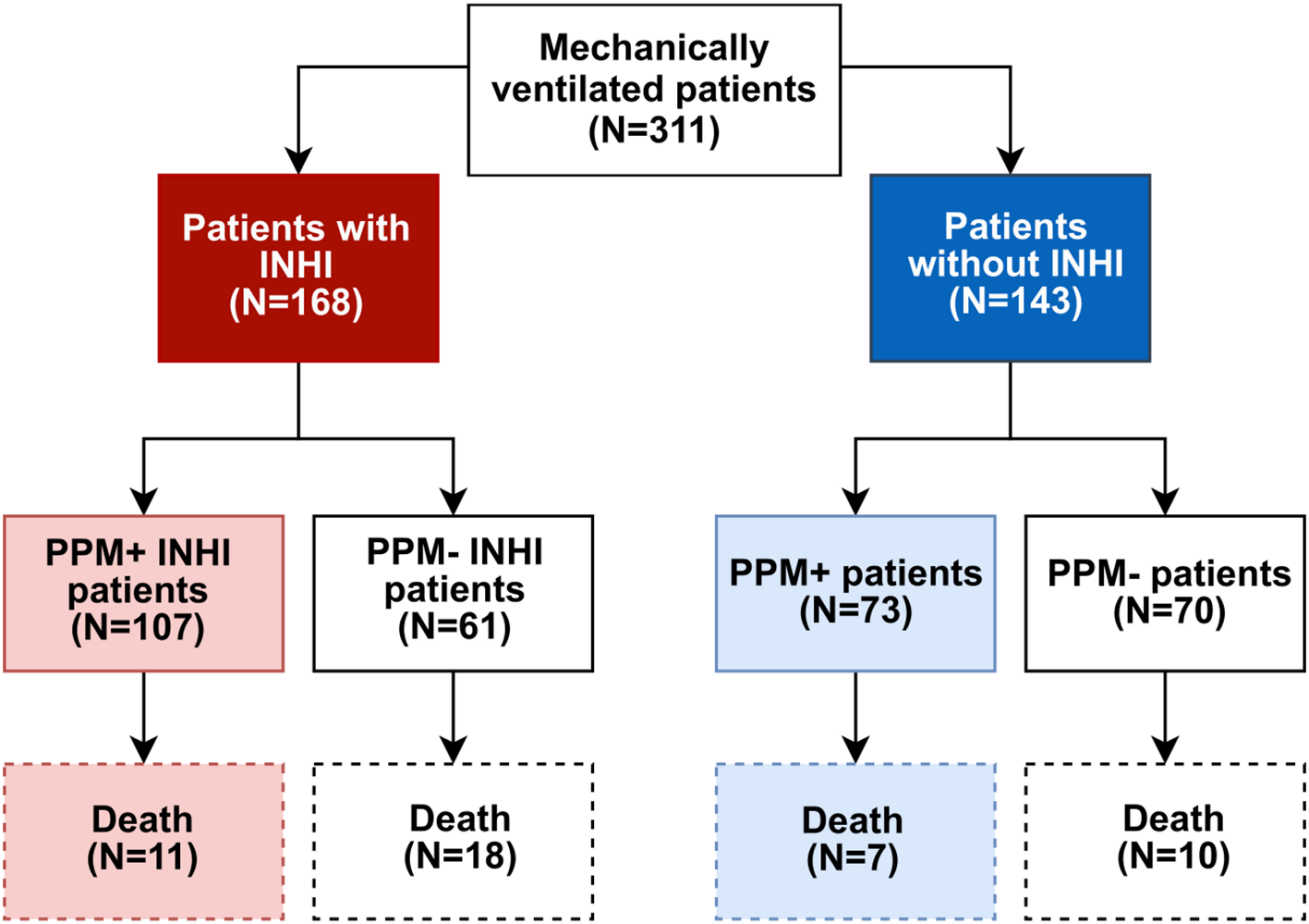
Flowchart of mechanically ventilated burn patients stratified by inhalation injury and PPM status, with subgroup sizes and mortality outcomes.

Of the total 311 patients hospitalized over the 15-year data collection period at the tertiary burn center who were mechanically ventilated for at least two days, 168 (54.0%) were diagnosed with INHI, while the remaining patients suffered burns without INHI. Detailed cohort categorization is shown in the flowchart in **Figure 1**; **Table 1**.

No statistically significant difference (*p* < 0.05) was observed between groups with and without INHI in any of the nine clinical variables studied (**Table 1**). Patients suffering from INHI were 1.68 times more likely to be PPM+ in the LRT during hospitalization (95% CI [1.07, 2.65]) than patients without INHI, but none of the studied biochemical markers were associated with the presence of INHI. The difference in ABSI scores between patients who died (median 11) vs. survivors (median 7.5) was statistically significant, as confirmed by the Wilcoxon rank-sum test (*p* < 0.001).

### 3.2 Microbiological findings

**Figure 2:**
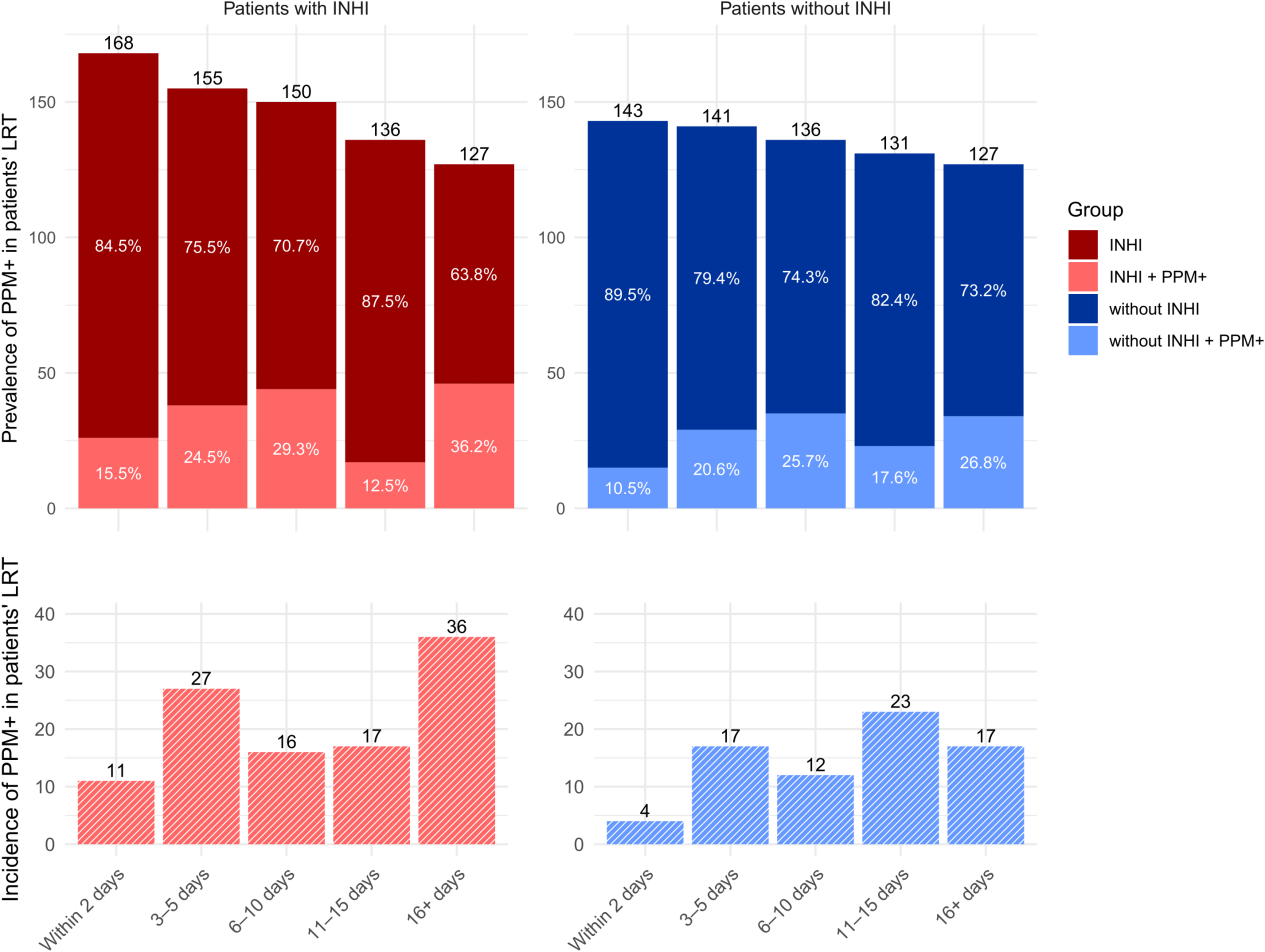
Prevalence and incidence of PPMs in LRT samples from patients over the course of hospitalization. PPM, potentially pathogenic microorganism; INHI, inhalation injury.

During hospitalization, LRT samples were PPM+ in total of 180 (57.9%) patients. Although some patients’ LRTs were colonized already on admission, prolonged hospitalization led to an increased incidence of positivity for PPMs (**Figure 2**). A similar trend was observed in both patients with and without INHI. By the end of the second week of hospitalization, a temporary decrease in the frequency of PPM+ patients was observed, but if hospitalization exceeded 16 days, the proportion of PPM+ patients increased again. However, since both groups had the same number of patients in the final hospitalization interval, the higher incidence of newly detected PPM positivity in the INHI group reflected a higher burden of microbial colonization potentially associated with INHI.

**Figure 3:**
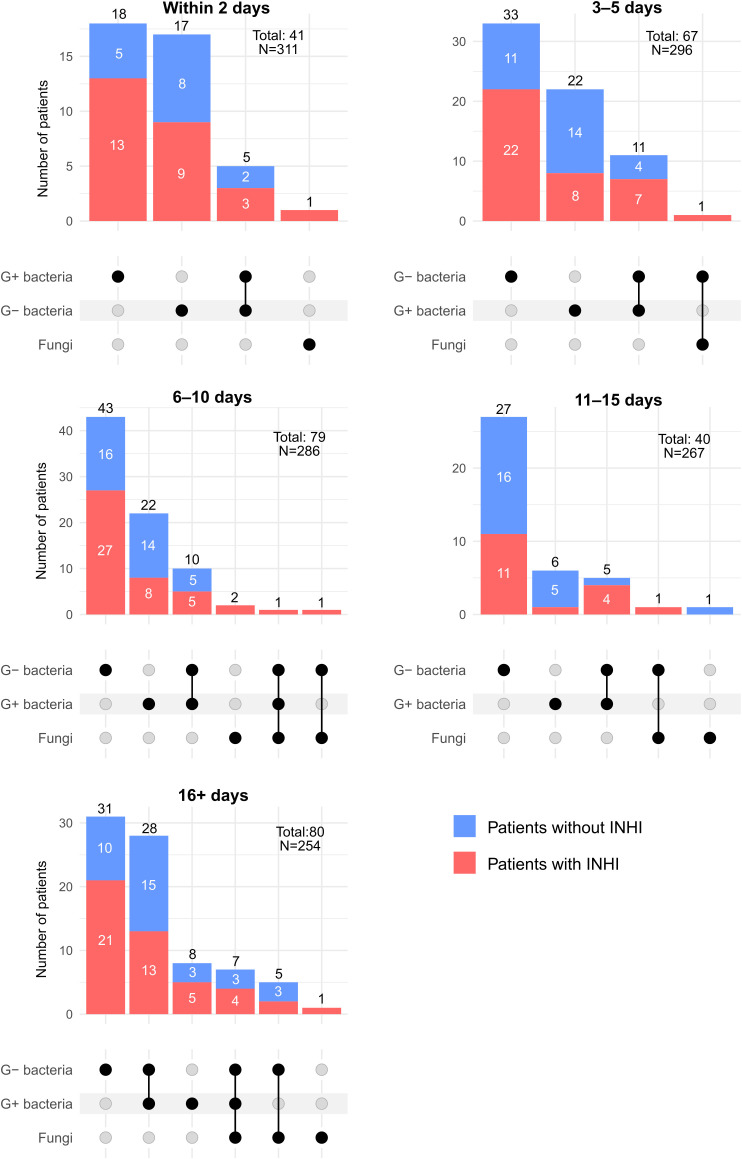
Upset plots of patients positive for individual PPM groups and their co-occurrences at individual time points. PPM+, positive for potentially pathogenic microorganisms; INHI, inhalation injury; LRT, lower respiratory tract.

The abundancies of G+ bacteria, G- bacteria, and microscopical fungi and their co-occurrence during hospitalization is presented in **Figure 3**. Although all three groups of PPMs were present in patients since the day of admission, G+ bacteria predominated at the beginning of the hospitalization.

Between Days 3 and 5 of hospitalization, the frequency of detecting G- bacteria in LRT samples significantly increased. A substantial increase in co-infections with G+ and G- bacteria was also observed, while the microscopical fungi appeared only once in that period.

Within the next investigated period (Days 6–10), the first sample containing representatives from all three groups of PPMs was detected. The presence of G- bacteria peaked within this period (55 patients, 19.2% of all patients hospitalized in that period). In other words, G- bacteria (most commonly *K. pneumoniae* and *P. aeruginosa*) were detected in 69.6% of PPM+ patients in this period.

Towards the end of the second week of hospitalization (Days 11–15), the frequency of PPM+ samples decreased (15.0%), returning nearly to the levels observed in the first two days (13.2%), see the upset plots in **Figure 3**.

**Table 2** presents the distribution of PPMs isolated from patients during hospitalization, categorized by the length of stay. Among G- bacteria, *Pseudomonas* sp. and *Enterobacter* sp. were the most commonly detected, with *Pseudomonas* sp. present in approximately one in six patients, especially in the period of 16+ days of hospitalization. In that period, G- bacteria from the genera *Pseudomonas*, *Enterobacter*, *Escherichia*, and *Acinetobacter* were already predominant. *Staphylococcus* spp. were the most common G+ bacteria at every time point. *Staphylococcus aureus* predominated in the early period, particularly within the first 10 days, whereas other *Staphylococcus* spp. became more frequent later during hospitalization, especially after Day 15. Methicillin-resistant *Staphylococcus aureus* (MRSA) was only identified in a single patient. Yeasts, particularly non-albicans *Candida*, showed a significant increase after 16 days of hospitalization, with *Candida glabrata* detected in the LRT of five patients, three of whom died. Among the twenty patients with at least one fungal isolate detected in the LRT, four died, corresponding to a mortality rate of 20% (see **Table 2**).

**Table 2:** PPMs isolated from LRT samples of patients during their hospitalization.

Microorganisms	Present at any time during hospitalization (N = 311)	Within 2 days (N = 311)	3–5 days (N = 296)	6–10 days (N = 286)	11–15 days (N = 267)	16+ days (N = 254)
G- bacteria	110	25	35	33	11	43
*Staphylococcus aureus*	59	15	27	17	3	9
Other *Staphylococcus* spp.^a^	42	1	1	9	5	31
*Streptococcus* sp.	19	11	5	4	1	1
*Enterococcus faecalis*	20	0	3	4	3	12
Other^b^	4	0	0	1	0	3
G- bacteria	151	23	46	55	33	71
*Klebsiella pneumoniae*	82	6	14	26	17	45
*Pseudomonas* sp.	48	0	3	8	4	40
*Enterobacter* sp.	33	2	7	8	6	14
*Escherichia coli*	29	6	11	10	4	5
*Acinetobacter* sp.	25	1	2	9	1	14
*Haemophilus* sp.	15	3	7	4	3	0
*Klebsiella oxytoca*	7	2	1	1	3	2
*Neisseria meningitidis*	6	6	1	1	0	0
*Proteus* sp.	5	1	2	0	1	1
Other^c^	18	2	6	5	0	8
Fungi	20	1	1	4	2	13
*Candida non-albicans*	11	0	0	2	1	8
*Candida albicans*	7	1	0	2	0	4
*Aspergillus* sp.	3	0	1	0	1	1

LRT, lower respiratory tract; PPMs, potentiallly pathogenic microorganisms.

^a^ Coagulase-Negative Staphylococci, *Staphylococcus haemolyticus*.

^b^ *Lactobacillus* sp., *Enteroccoccus faecium*, *Propionibacterium* sp.

^c^ *Stenotrophomonas maltophilia*, *Serratia marcescens*, *Aeromonas* sp., *Citrobacter* sp., *Morganella morganii*, *Moraxella catarrhalis*, *Pantoea agglomerans*, *Prevotella melaninogenica*, *Providentia stuartii*, *Raoultella ornithinolytica*.

#### 3.2.1 Extended-spectrum β-lactamase producers

Several of the detected G- bacteria were ESBL producers (i.e., strains resistant to multiple antibiotics), with *K. pneumoniae* being the most common (53 patients). Other ESBL producers included *Enterobacter cloacae* (7 patients), *Escherichia coli* (1 patient), and *Acinetobacter* sp. (1 patient). Out of the total 87 K*. pneumoniae* infections, 53 were ESBL producers, emphasizing the significant role of *K. pneumoniae* in ESBL-related infections.

The relationship between the presence of ESBL-producing bacteria and mortality was assessed by comparing groups of patients (i) positive for ESBL-producing bacteria and (ii) positive for non-ESBL producing bacteria. In the latter group, 8 patients out of 122 died, while in the group positive for ESBL-producing bacteria, 10 out of 58 patients died (*p* = 0.049, Pearson’s chi-squared test), indicating that ESBL production was associated with a higher likelihood of death. The OR was 2.97, suggesting that the odds of dying were almost three times higher in patients with ESBL-producing bacteria compared to those with non-ESBL producing bacteria.

Also, a significant difference in median hospital stay length between patients positive for ESBL-producing bacteria (48.0 days) and those with non-ESBL-producing bacteria (35.5 days) was detected (*p* < 0.001, Mann-Whitney test), indicating that the presence of ESBL-producing bacteria in LRT samples was associated with longer hospital stays.

There was no statistically significant association between INHI and the presence of ESBL-producing PPMs+ nor between INHI and other G- bacteria. However, given the relatively low number of patients with ESBL-producing bacteria in our study, the results need to be considered with care and no strong conclusions can be drawn from this particular subanalysis.

### 3.3 Survival analysis

A survival multistate analysis was performed to determine the probability of being in a specific state (at risk, discharged from treatment – censoring event, PPMs positive or dead) at given time. In the cohort of mechanically ventilated patients (N = 311), 180 patients developed PPM positivity during their hospital stay, and 18 of these subsequently died. Another 28 deaths occurred in patients without any PPMs positivity in LRT samples (**Figure 1**). The mean restricted event-free time (i.e., time until PPM positivity in LRT or death) was 32 days of hospitalization in patients with INHI and 64 days in those without INHI, indicating that INHI is a risk factor for earlier event occurrence.

Notably, there was a significant difference in the average length of hospital stay between two groups: patients who were infected and died spent an average of 23.7 days in the hospital, whereas those who died without of any PPMs presence had an average stay of 6.2 days. The median time to first acquisition of PPMs for the entire dataset was 9.0 days.

**Figure 4:**
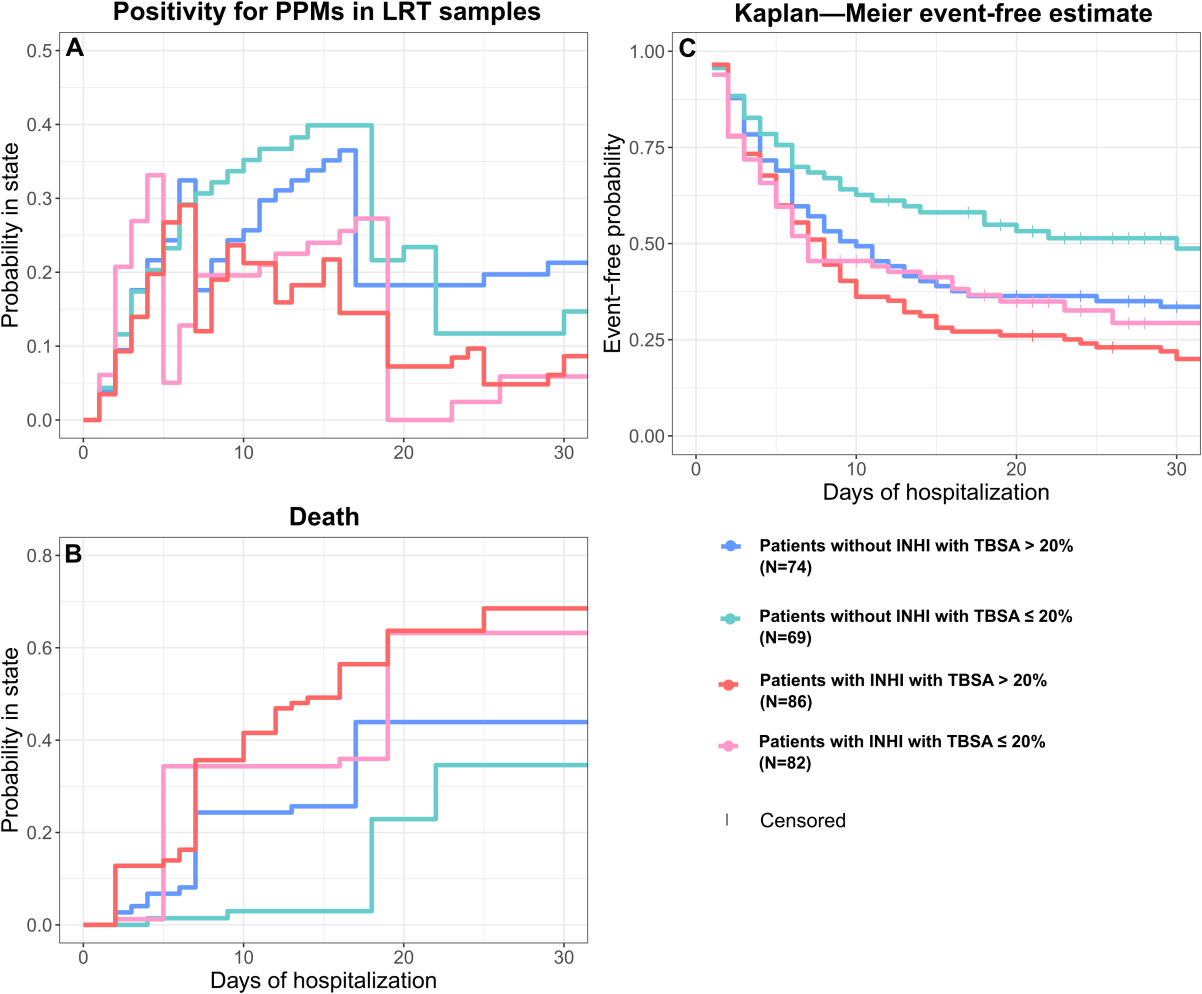
Kaplan–Meier estimate and cumulative incidence curves of multistate survival analysis. **(A)** Cumulative incidence curves and Kaplan–Meier estimates for the probability of not experiencing the events, comparing four groups. Patients were divided into with INHI and without INHI groups, and further stratified by TBSA ≤ 20% and > 20%. The Kaplan–Meier curve estimates the likelihood of remaining free from the event over time, taking into account that some patients may leave the study early or have incomplete follow-up [censoring; panel **(C)**]. The cumulative incidence curves illustrate the risk of experiencing PPM positivity [panel **(A)**] or death [panel **(B)**]. Specifically, they represent the proportion of patients who developed the respective event among those still at risk at each time point. For PPMs positivity, patients who died before PPM occurrence were considered as having experienced a competing event. Note: INHI, inhalation injury; PPM, potentially pathogenic microorganism.; LRT, lower respiratory tract; TBSA, total burned surface area.

In this analysis, a decrease in the cumulative incidence of PPM positivity occurred because some patients died before becoming PPM positive, removing them from the risk set and thus lowering the observed incidence of PPM positivity over time (**Figure 4**, panel A). In our analysis, we focused on the first 30 days of hospitalization, as this was the period when most events occurred.

Until Day 5, groups showed a steep increase in PPM positivity (**Figure 4**, panel A); after this period, however, the mortality of PPM+ INHI patients (11 PPM+ INHI patients out of 18 in total died eventually, 61.1%) caused a change in this trend (**Figure 4**, panel B). At 30 days of hospitalization, the probability of remaining event-free among patients with INHI with TBSA > 20% (20.0%) and patients with INHI with TBSA ≤ 20% (29.3%), was lower than in patients without INHI with TBSA > 20% (33.6%) (**Figure 4**, panel C). A similar pattern was observed for mortality: patients with INHI with TBSA > 20% (68.5%) and patients with INHI with TBSA ≤ 20% (63.2%), had a substantially higher probability of death compared to patients without INHI with TBSA > 20% (43.9%) (**Figure 4**, panel B). Overall, patients with INHI exhibited higher risks of adverse outcomes compared to those without INHI regardless of TBSA.

Given the approximately proportional distribution of patients across these groups and the comparable median TBSA between patients with and without INHI, we simplified the model into two groups based on the presence or absence of INHI (**Supplementary Figure S1**). At 30 days of hospitalization, the probability of remaining event-free was 25.9% (95% CI [19.5, 34.4]) for patients with INHI, compared to 42.4% (95% CI [34.7, 51.6]) for those without. The time to first event differed significantly between these groups, with patients suffering from INHI facing a higher risk of complications or death within the first 30 days (*p* = 0.002, log-rank test; **Supplementary Figure S1**).

**Figure 5:**
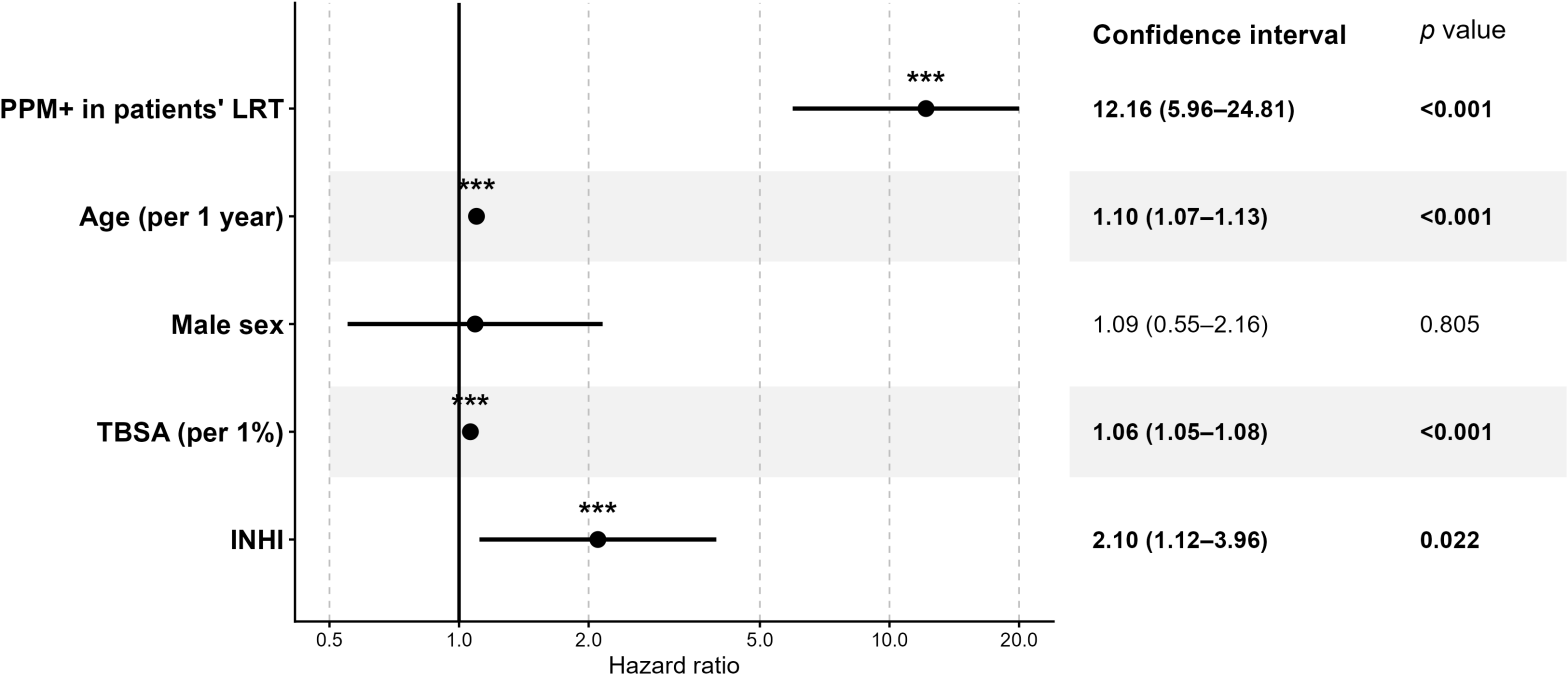
Forest plot of hazard ratios for in-hospital mortality from the time-dependent Cox proportional hazards model. INHI, inhalation injury; PPM, potentially pathogenic microorganism.; LRT, lower respiratory tract; TBSA, total burned surface area; ***suggesting *p* < 0.05.

However, as the Kaplan-Meier analysis is not adjusted for confounders, this analysis should be considered exploratory only. For this reason, and to further examine whether the occurrence of PPM positivity during hospitalization was associated with subsequent in-hospital mortality, we fitted a Cox proportional hazards model with PPM+ treated as a time-dependent exposure. Based on the sensitivity analysis, the model was subsequently modified by excluding four influential patients (N = 307). In this model, the occurrence of PPM+ was associated with a markedly increased subsequent hazard of in-hospital death [HR 12.16, 95% CI (5.96, 24.81); *p* < 0.001]. Higher age [HR 1.10 per year, 95% CI (1.07, 1.13); *p* < 0.001], larger TBSA [HR 1.06 per 1% increase, 95% CI (1.05, 1.08); *p* < 0.001], and INHI [HR 2.10, 95% CI (1.12, 3.96); *p* = 0.022] were also associated with increased in-hospital mortality after adjustment for the other covariates, whereas sex was not (male vs. female: HR 1.09, 95% CI [0.55, 2.16]; *p* = 0.805) (**Figure 5**).

The modified model showed an acceptable overall fit. No evidence of problematic collinearity among age, sex, TBSA, and INHI was identified; the correlation between age and TBSA was weak, and adjusted GVIF values were close to 1 for all covariates. The global test did not indicate a significant violation of the proportional hazards assumption (*p* = 0.148), and no relevant non-proportionality was observed for the remaining covariates. Residual plots did not show any major problems.

## 4 Discussion

### 4.1 Insights into demographic and epidemiological patterns

In our study, INHI was diagnosed in 54.0% of all patients admitted to the Burn ICU between 2007 and 2022, a proportion comparable to that reported in a smaller Romanian study investigating patients admitted to the ICU in 2021 (60.2%) (Niculae et al., 2023). More than 30 years in the past, it was reported that INHI patients generally have more extensive burns than those without INHI (with INHI ~39.7% and without INHI ~12.2%) (Tredget et al., 1990) and this has been expected in our study. However, our results showed no statistically significant difference in TBSA between the two patient groups (with median TBSA of 27.0% and 26.6% with and without INHI, respectively). This could be also associated with the progress in INHI diagnosis over the last decades.

INHI is generally known to significantly increase the mortality of burn patients up to 45% (Tredget et al., 1990; Cancio, 2005; Rehberg et al., 2009; Woodson, 2009; Walker et al., 2015; Walsh et al., 2017; Dyamenahalli et al., 2019). In our study, the hospital mortality rate of patients with INHI was 17.3%, which is comparable to the 12.1% observed in a smaller study (58 patients with INHI between 2008 and 2011) (Liodaki et al., 2014). In contrast, a significantly higher mortality rate of 31% was previously reported among INHI patients with cutaneous fire/flame burns admitted to a burn center intensive care unit (Witt et al., 2021).

In our study, the presence of INHI did not significantly impact the mean duration of hospitalization, which was nearly identical in both groups (40.9 days for patients without INHI vs. 39.5 days for those with INHI). This, however, contrasts with findings from another study (reporting a similar mortality rate), where hospitalization durations were shorter (27.5 days for patients with INHI vs. 16 days for those without INHI) (Liodaki et al., 2014). We hypothesize that the observed difference in hospitalization duration is more likely attributable to the positivity for PPMs in LRT samples. This is consistent with the previous studies associating prolonged hospitalization with the development of nosocomial infections (Oncul et al., 2002; Guo et al., 2018; Corcione et al., 2020).

### 4.2 Presence of PPMs in LRT samples and trends over time

INHI poses a substantial risk for subsequent respiratory infections due to direct damage to the airway epithelium and compromised mucociliary clearance mechanisms, which are critical in preventing pathogen colonization (Melo-González et al., 2022). Furthermore, the systemic inflammatory response triggered by INHI can lead to immunosuppression, further exacerbating vulnerability to opportunistic infections (Lipovy et al., 2024). It was previously reported that pneumonia occurred in 38% of patients with INHI (Shirani et al., 1987). Further, multiple studies have shown that up to 65% of deaths in burn victims are attributable to infection (Sharma et al., 2006; Bloemsma et al., 2008; Keen et al., 2010; Krishnan et al., 2013; Lachiewicz et al., 2017). The percentage of LRT PPM+ hospitalized patients increased over the first 10 days of hospitalization in our study (27.6% overall in the period of Days 6-10). This was followed by a notable drop in the second week of hospitalization, likely due to the administration of antimicrobials. After two weeks of hospitalization, however, the percentage of LRT PPM+ patients climbed again, peaking at 31.5%.

Infections in patients with INHI are typically caused rather by bacteria than by microscopical fungi. Microbial shift from G+ to G- bacterial species over the hospitalization period observed in our study (especially after the second week of hospitalization) is consistent with previously reported findings (Lachiewicz et al., 2017; Corcione et al., 2020; Pu et al., 2020). In line with previous studies (Taneja et al., 2013; Wanis et al., 2016; Chong et al., 2018; Vinaik et al., 2019), *K. pneumoniae* (G-), *S. aureus* (G+), and *P. aeruginosa* (G-) were the most common pathogens in LRT samples also in our study, regardless of the presence of INHI. A previous study reported predominance of G- bacteria in INHI patients and G+ bacteria in those without (Chong et al., 2018); however, our data did not confirm this pattern.

Although isolated fungal infections are uncommon, fungal pathogens must not be neglected. *Candida* sp. (mostly by *Candida albicans*) and *Aspergillus* sp. were the most common fungal PPMs detected in our cohort. This is not surprising as these two microscopical fungi are the most abundant species in airways of patients critically ill from other causes (Huerta et al., 2014; Su et al., 2015; Martinsen et al., 2021).

#### 4.2.1 ESBL-producing PPMs

The presence of ESBL-producing bacteria in the LRT poses a significant and escalating challenge in clinical practice, particularly in hospitalized and critically ill patients. These formidable pathogens, predominantly *E. coli* and *K. pneumoniae*, are a major concern due to their resistance to commonly used antibiotics (Sivaraman et al., 2021; Husna et al., 2023). The clinical impact of ESBL-producing bacteria is substantial, leading to poorer patient outcomes, increased healthcare burdens, and significant challenges for clinicians (Bennett et al., 2010). Our findings demonstrated an association between ESBL-producing PPMs isolated from the LRT and longer hospital stays compared to non-ESBL PPMs (median 48 vs. 35.5 days in our cohort, *p* < 0.001). A similar association between ESBL production and prolonged hospitalization has been reported before (Loh et al., 2007) in a study focusing exclusively on *K. pneumoniae*, further reinforcing the relevance of this observation in specific PPMs. This aligns with previous studies (Vance et al., 2023; Handal et al., 2024), which also reported prolonged hospital stays for patients with LRT infections caused by ESBL-producing PPMs.

Evidence on the prognostic impact of ESBL-producing pathogens in lower respiratory tract samples among mechanically ventilated burn patients is mixed. While another study on this topic reported no significant mortality difference between ESBL and non-ESBL isolates (Bennett et al., 2010), our results showed nearly threefold higher odds of mortality in the ESBL+ group.

### 4.3 Survival analysis and key predictors

INHI is one of the most critical factors influencing mortality in burn patients, often increasing the risk of death significantly (El-Helbawy and Ghareeb, 2011). LRT infections remain the most common complications of INHI and at the same time, also have the most serious clinical consequences (Pu et al., 2020). Hence, this complication is undeniably responsible for the mortality rate. The mean time to death without PPM+ was 6.2 days, whereas the mean time to first PPM+ detection was 9.0 days, suggesting a temporal separation between these events. Specifically, deaths without prior PPM+ tended to occur earlier in the hospitalization course, while PPM+ detection was more frequently observed among patients with longer in-hospital survival. Our epidemiological and microbiological data revealed that LRT PPM+ patients who died spent an average of 23.7 days in the hospital. This is consistent with previous findings, where most deaths typically occurred within a few weeks following hospital admission (Yoneda et al., 2024; Karikari et al., 2025). In our cohort, most events also occurred within the first 30 days of hospitalization, reinforcing the acute nature of INHI-related complications. Notably, both groups experienced a sharp rise in PPM+ up to Day 8; however, this trajectory was subsequently altered due to high early mortality among PPM+ INHI patients (11 out of 18; 61.1%) (Chong et al., 2018), which reduced the number of individuals remaining at risk for further PPM +. In contrast, the slower rise in LRT PPM+ non-INHI patients continued through Day 16. By Day 30, the probability of remaining event-free was markedly lower in INHI patients compared to those without INHI (25.9% vs. 42.4%; *p* = 0.002, log-rank test).

These descriptive survival findings were further strengthened by the time-dependent Cox analysis. After adjustment for age, sex, TBSA, and INHI, the occurrence of PPM+ during hospitalization remained associated with a markedly increased subsequent hazard of in-hospital death. This is broadly consistent with previous reports identifying INHI as a major determinant of poor outcomes in burn patients and showing that pulmonary infectious complications may contribute to increased morbidity and, in some studies, mortality in this population (Shirani et al., 1987; Tredget et al., 1990; El-Helbawy and Ghareeb, 2011; Chong et al., 2018; Witt et al., 2021; Charles et al., 2022). At the same time, the timing of PPM+ occurrence during hospitalization needs to be considered, because a positive LRT culture can only be detected in patients who survive long enough for this finding to occur.

In some previous studies, pneumonia or other pulmonary complications were associated mainly with longer ventilation and prolonged hospitalization, but not always with mortality after multivariable adjustment (Charles et al., 2022; Johnson et al., 2025). In contrast, our time-dependent analysis focused on the first positive LRT culture during hospitalization and showed that once PPM+ occurred, the subsequent hazard of in-hospital death was substantially higher, even after accounting for other major clinical factors. This interpretation is also in line with multistate analyses showing that hospital-acquired infection is associated with increased mortality when the timing of infection is explicitly incorporated into the model (Guo et al., 2018).

### 4.4 Limitations

Our study had some limitations. First, the data on antimicrobial use were not collected in this study, precluding the possibility of analyzing their effect on the dynamics of the individual microbial groups and on the treatment outcome. Second, there is inconsistency in the diagnosis and treatment of INHI among centers. In our study, however, we aimed to acquire highly accurate classification of INHI, which was ensured using bronchoscopy or laryngoscopy in all patients (Marek et al., 2007). From this perspective, the monocentric design of our study supporting consistency in diagnosis can be actually considered a strength of the study. Third, in the study, we only analyzed the dynamics of microbiological findings without associating them with clinical picture of LRTI. Still, our paper provides an analysis of the associations between INHI and the PPM positivity, an aspect not addressed in any study so far.

## 5 Conclusion

INHI remains one of the critical determinants of both clinical management and mortality outcomes in burn patients. It is also a principal risk factor for rapid onset of PPM positivity and LRTI. Our analysis demonstrates that patients with INHI experience significantly accelerated timelines to adverse endpoints, including PPM positivity and mortality. The predominance of multidrug-resistant bacterial strains among isolated PPMs is of particular concern, representing a substantial therapeutic challenge. These findings suggest that aggressive and preemptive management protocols are essential in patients with INHI. Clinicians should be particularly vigilant for signs of infection and consider the increased risk of multidrug-resistant PPMs when selecting empirical antimicrobial therapies in this patient cohort.

## Data Availability

The data analyzed in this study is subject to the following licenses/restrictions: no restrictions. Requests to access these datasets should be directed to raska.filip@fnbrno.cz.
