# Supplementary material for: Epidemiology of microbiological findings in the lower respiratory tract in mechanically ventilated patients with and without inhalation injury
Source: Front Cell Infect Microbiol. 2026 May 13;16:1789404. doi: 10.3389/fcimb.2026.1789404 (PMC13212340; doi:10.3389/fcimb.2026.1789404)
Supplement: Supplementary file 1 [file DataSheet1.pdf]

Supplementary Material

Figure S1. Kaplan-Meier estimate and cumulative incidence curves of multistate survival analysis.

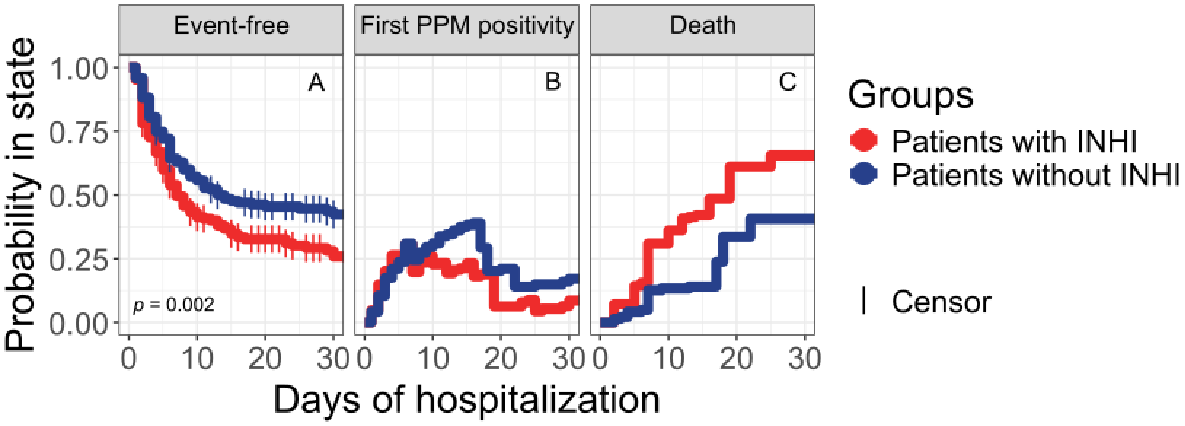

Note: INHI, inhalation injury; PPM, potentially pathogenic microorganism. The *p* refers to the log-rank test.
